# Bioaccumulation and Toxicity of Carbon Nanoparticles Suspension Injection in Intravenously Exposed Mice

**DOI:** 10.3390/ijms18122562

**Published:** 2017-11-29

**Authors:** Ping Xie, Sheng-Tao Yang, Tiantian He, Shengnan Yang, Xiao-Hai Tang

**Affiliations:** 1 State Key Laboratory of Oral Diseases, West China College of Stomatology, Sichuan University, Chengdu 610041, China; xieping318@aliyun.com; 2 College of Chemistry & Environment Protection Engineering, Southwest Minzu University, Chengdu 610041, China; 18641460881@163.com; 3 Chongqing Lummy Pharmaceutical Co., Ltd., Chongqing 401123, China; tthe2017@126.com

**Keywords:** carbon nanoparticles suspension injection, biodistribution, biosafety, Raman spectroscopy, nanotoxicity

## Abstract

Carbon nanoparticles suspension injection (CNSI) has been widely used in tumor drainage lymph node mapping, and its new applications in drug delivery, photothermal therapy, and so on have been extensively investigated. To develop new clinical applications, the toxicity of CNSI after intravenous exposure should be thoroughly investigated to ensure its safe use. Herein, we studied the bioaccumulation of CNSI in reticuloendothelial system (RES) organs and the corresponding toxicity to mice. After the intravenous injection of CNSI, no abnormal behavior of mice was observed during the 28-day observation period. The body weight increases were similar among the exposed groups and the control group. The parameters of hematology and serum biochemistry remained nearly unchanged, with very few of them showing significant changes. The low toxicity of CNSI was also reflected by the unchanged histopathological characteristics of these organs. The injection of CNSI did not induce higher apoptosis levels either. The slight oxidative stress was observed in RES organs at high dosages at day 7 post-exposure. The implication to the clinical applications and toxicological evaluations of carbon nanomaterials is discussed.

## 1. Introduction

Carbon nanomaterials, such as carbon nanoparticles, carbon quantum dots, fullerene, carbon nanotubes (CNTs), and graphene, have attracted great research interest in the past decades [1,2]. The unique structures and fantastic properties make carbon nanomaterials suitable for biomedical applications [3]. In particular, carbon nanomaterials have been found to have great potential in theranostics, including bioimaging, diagnosis, drug delivery, gene therapy, photothermal therapy, and so on [4,5,6]. For example, carbon quantum dots could be used for sentinel lymph node imaging and photodynamic therapy [7,8]. Graphene-based drug delivery systems have been used in cancer treatment [9]. The laboratory results show the bright future of carbon nanomaterials in clinical applications. Although more efforts are required to commercialize them, carbon nanomaterials are expected to benefit public health in the future.

Among these novel carbon nanomaterials, carbon nanoparticles suspension injection (CNSI) is the only one that has been produced on a large scale and applied in clinical treatments. Annually, over 100,000 patients receive CNSI injections during oncological surgery, and the numbers keep increasing quickly [10]. Currently, CNSI is only used for the lymphatic mapping and for distinguishing the parathyroid gland [11,12,13,14]. First, CNSI migrates fast in lymphatic vessel and is trapped in lymph nodes to stain them black. Researchers have used diverse cancer models to establish the high performance of CNSI in tumor drainage lymph node (TDLN) mapping. For instance, Li et al. dissected the lymph nodes after staining with CNSI in advanced gastric cancer [11]. Wu et al. achieved the TDLN mapping of early breast cancer by using CNSI [12]. Zhu et al. performed central lymph node dissection for patients with papillary thyroid carcinoma using CNSI as the tracer [13]. Second, CNSI does not stain the parathyroid gland during thyroid carcinoma surgery, thus reducing the risk of false resection. Gu et al. used CNSI to identify parathyroid from thyroid and lymph nodes during surgery, which largely preserved the parathyroid glands from false resection [14].

It should be noted that CNSI shares many characteristics with other carbon nanomaterials, so CNSI might be used in more biomedical areas beyond the mapping of lymph nodes. In fact, CNSI adsorbs drugs well and could be used as a drug carrier. For example, Xie et al. found that CNSI adsorbed epirubicin and doxorubicin efficiently [15]. Yang et al. reported that CNSI significantly enhanced the drug concentration in lymph nodes and reduced the plasma drug concentration in the regional lymphatic chemotherapy of epirubicin [16]. Other potential applications of CNSI might include photothermal therapy, gene delivery, and use as an immune adjuvant. However, before new explorations and applications are proposed, the toxicity of CNSI is an urgent issue that needs to be addressed [17,18]. Because the previous applications of CNSI only focused on regional injection, the toxicity of CNSI has not been evaluated upon exposure through other pathways. For clinical applications, the most important exposure is intravenous injection. Unfortunately, the biosafety information of intravenously exposed CNSI is not available to date.

Although the toxicity of CNSI is unknown, the literature results of other carbon nanomaterials indicate that the toxicity of carbon nanomaterials is generally low after intravenous injection and the properties of carbon nanomaterials regulate their biosafety [19,20]. Previously, we reported that CNTs accumulated in body during the 90-day observation period and only induced slight toxicity and oxidative stress [21]. Another similar formulation to CNSI was carbon quantum dots (polyethylene glycol (PEG) functionalized carbon nanoparticles), which were nontoxic to mice after intravenous exposure [22]. In other reports, some carbon nanomaterials were found to be toxic. For examples, graphene oxide (GO) was found to induce macrophage nodule formation in the lungs after intravenous injection at 2.1 mg/kg bodyweight [23]. After dextran functionalization, the tolerable dose increased to 125 mg/kg, and toxicity was observed only at 250 mg/kg or higher [24]. Separately, Zhang and co-workers found that CNTs induced hepatotoxicity to mice after intravenous injection in a 2-month investigation [25]. Therefore, it is very likely that CNSI has low toxicity, but this hypothesis requires more evidence.

In this study, we systematically investigated the biodistribution and toxicity of CNSI in mice after intravenous injection. The bioaccumulation of CNSI was studied by using Raman spectroscopy and optical microscopy. The behaviors were recorded and the body weights were measured. The hematology and serum biochemistry were analyzed to reveal potential function changes. The histopathological changes were investigated under an optical microscope. The apoptosis of tissues was assayed by the terminal deoxynucleotidyl transferase-mediated dUTP nick-end labeling (TUNEL) method. The oxidative stress was also measured to reveal the possible toxicological mechanism. The implications to the biosafety evaluations and the new applications of CNSI are discussed.

## 2. Results and Discussion

### 2.1. Characterization of CNSI

**Figure 1:**
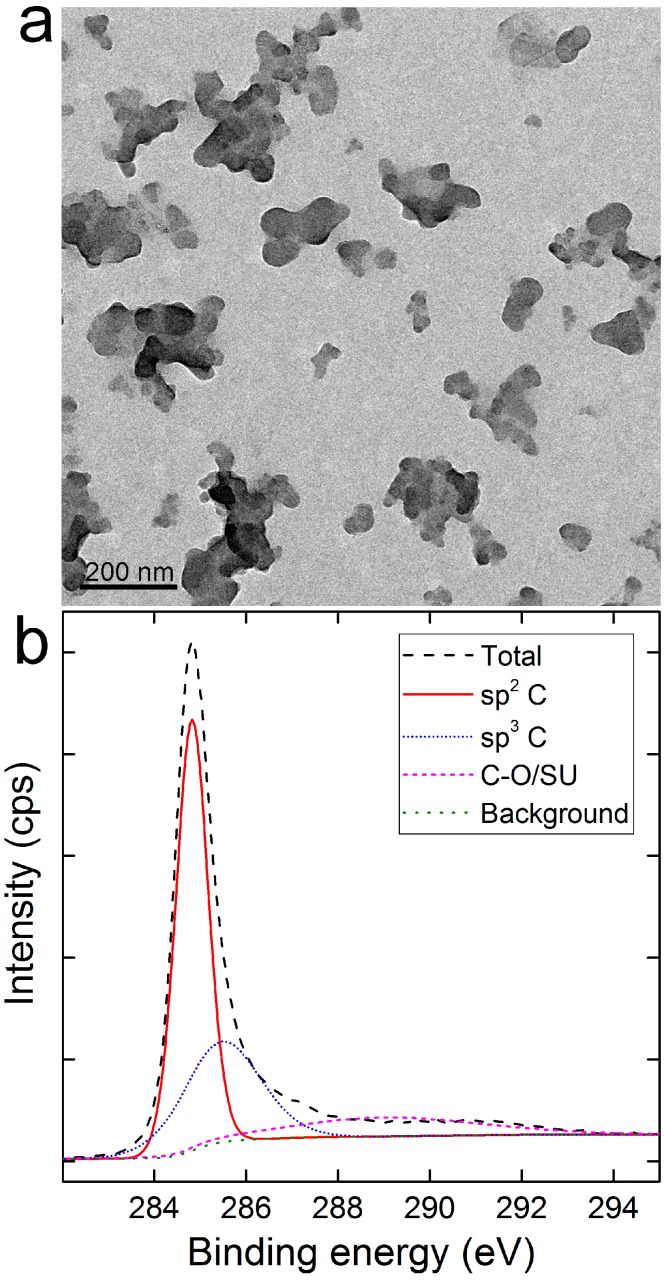
Transmission electron microscopy (TEM) image (**a**) and C1s X-ray photoelectron spectroscopy (XPS) spectrum (**b**) of carbon nanoparticles suspension injection (CNSI).

According to the preparation procedure of CNSI, carbon black (CH40, Mitsubishi Chemical Co., Tokyo, Japan) containing carbon nanoparticles (~21 nm in diameter) was washed by ethyl acetate and nitric acid before dispersion in polyvinyl pyrrolidone (PVP). CNSI was a dark black dispersion of carbon nanoparticles that remained stable for months of storage at room temperature. As indicated in Figure 1a, CNSI was composed of small carbon particles, revealed under transmission electron microscopy (TEM, Autoflex, Bruker, Bonn, Germany). The diameters of these particles were in the range of 10–50 nm, which further aggregated into larger aggregates. According to the dynamic light scattering (DLS) analyses, CNSI had a hydrodynamic radius of 189 nm, reflecting the aggregation of carbon nanoparticles. The chemical components and functional groups of carbon nanoparticles without adding suspending reagent PVP were characterized by X-ray photoelectron spectroscopy (XPS) and infrared spectroscopy (IR). There were 93.8 at % of carbon atoms in the carbon core of CNSI. Other elements included 5.0 at % O and 1.2 at % N. According to the C1s XPS spectrum, 54.6% of the carbon atoms were sp^2^ carbon. The other carbon atoms were 30.9% of sp^3^ carbon and 14.5% of C–O bond or shake-up signal [17]. For the broad band at 289.0 eV, it was assigned to C–O and other shake-up components, resulting in an extremely broad full width at half maximum (FWHM). It should be noted that XPS only detected the signals of the surficial atoms (several nanometers in depth). Other methods such as energy dispersive spectroscopy and elementary analysis are also recommended for future studies. The IR analysis indicated the presence of –OH (3440 cm^−1^), aromatic C–C (1640 cm^−1^), and 1090 cm^−1^ (C–O). No signal was observed at around 1730 cm^−1^, indicating the lack of C=O. Overall, the characterization data suggested the CNSI sample contained no toxic impurity and was properly oxidized for dispersing, thus suitable for the following distribution and toxicity assays.

### 2.2. Bioaccumulation of CNSI in Reticuloendothelial System (RES)

**Figure 2:**
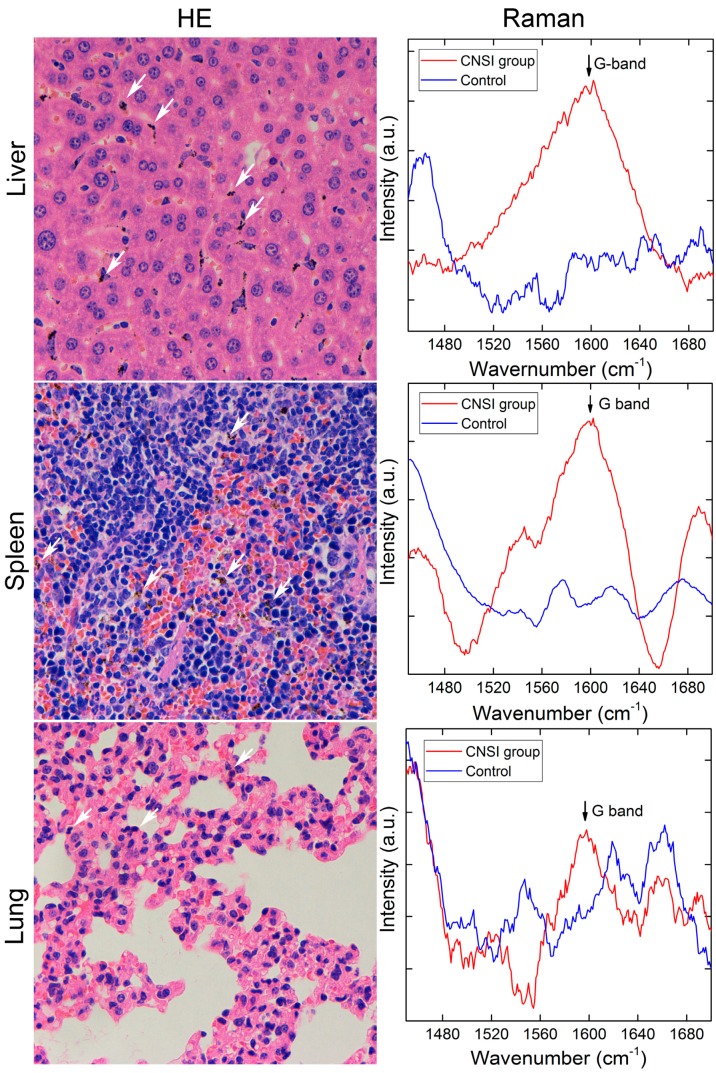
Bioaccumulations of CNSI in reticuloendothelial system (RES) organs, including the liver, spleen, and lungs. The dark aggregates of CNSI are indicated by white arrows (400×).

According to the literature, in many cases, carbon nanomaterials were trapped in the liver, spleen, and lungs after intravenous injection [20,26]. Small particles of carbon nanomaterials might also accumulate in kidneys for renal excretion [22,27]. Here, we checked the hematoxylin-eosin (HE) staining slides of the heart, liver, spleen, kidneys, lungs, and axillary lymph node. As shown in Figure 2, many large dark brown particles were observed in the liver (marked by white arrows). These large aggregates were too big for cellular uptake and most likely existed in the intercellular space. The smaller ones might enter the Kupffer cells [26]. The particles were smaller in the spleen, but still easy to recognize. For the lungs, only a very faint brown color was distinguished after careful checking. The brown colored spots were assigned to CNSI aggregates, which were not found in the control group. In the other three organs, no brown colored spots were found, suggesting the absence of CNSI. To confirm the accumulation of CNSI in mice, the grounded tissue samples were analyzed by Raman spectroscopy, in which the G band at around 1590 cm^−1^ was a typical signal of sp^2^ carbon structure. It should be noted that CNSI had a highly disordered structure, so the G band was much weaker than other typical sp^2^ carbon nanomaterials, such as CNTs and graphene. The 785 nm laser was used to avoid the strong autofluorescence of tissues. Clearly, G-band signals were found in the liver, spleen, and lungs. Consistent with the hemoglobin (HE) staining, stronger signals were found in the liver and spleen, while a very weak signal was observed for the lungs (slightly higher than the background signal). Nevertheless, the results of optical microscopy and Raman spectroscopy collectively indicated the accumulation of CNSI in RES organs. According to the literature, carbon nanomaterials are easily bound with proteins in the bloodstream [19]. Thus, CNSI might be recognized by opsonin after entering the blood circulation, where opsonization led to the capture in RES organs [28]. Most of the CNSI particles would likely be trapped by phagocytic cells, e.g. Kupffer cells [26], and the extremely large aggregates were stopped in the intercellular space. The very low pulmonary accumulation indicated that CNSI was well dispersed in the blood circulation to escape the filtration by the pulmonary capillary [26], which was consistent with the observations that CNSI dispersed well in water, saline, serum, and cell culture medium. The RES accumulations also reminded us to investigate the potential toxicity of CNSI to the accumulating organs. In addition, no CNSI was detected in other tissues or excreta by Raman spectroscopy due to the lower sensitivity comparing to other quantitative methods, such as isotope labeling and fluorescence imaging [26].

### 2.3. Toxicity Evaluations

During the 28-day observation period, no mouse died and no obvious abnormal behavior was observed. The body weight increases were similar (*p* > 0.05) among the control group and the CNSI-exposed groups (Table 1). The normal behaviors and body weight increases suggested that CNSI had low toxicity to mice after intravenous injection. This was consistent with the clinical observations that only several cases in all the treated patients (over 500,000) showed very short hyperpyrexia after the regional injection of CNSI [10]. The low toxicity of CNSI was then verified by the assays of hematology, serum biochemistry, histopathology, and apoptosis.

First, the hematological parameters were analyzed after exposure to CNSI. The data are listed in Table 2. At 1 day post-exposure, most parameters remained normal after the intravenous injection of CNSI. Significant changes were only observed for hemoglobin (HB) and mean corpuscular volume (MCV). The HB increased from 145 g/L (control group) to 155 g/L (80 µg group) and 156 g/L (320 µg group). The MCV increased from 49 fL (control group) to 56 fL (80 µg group). Even when the changes were statistically significant, the increases were generally small. Therefore, the hematological toxicity of CNSI was negligible at 1 day post-exposure. The situations were similar for the data at 7 days and 28 days, and only one or two datum points showed statistical changes. At 7 days, the mean corpuscular hemoglobin (MCHC) and MCV values of the 320 µg group were slightly larger than those of the control group. At 28 days, only the mean platelet volume (MPV) of the 160 µg group was higher than that of the control group. No apparent dose-dependent or time-dependent trends were observed for these changes. Thus, the hematology indicated that CNSI was nearly nontoxic to mice after intravenous injection.

**Figure 3:**
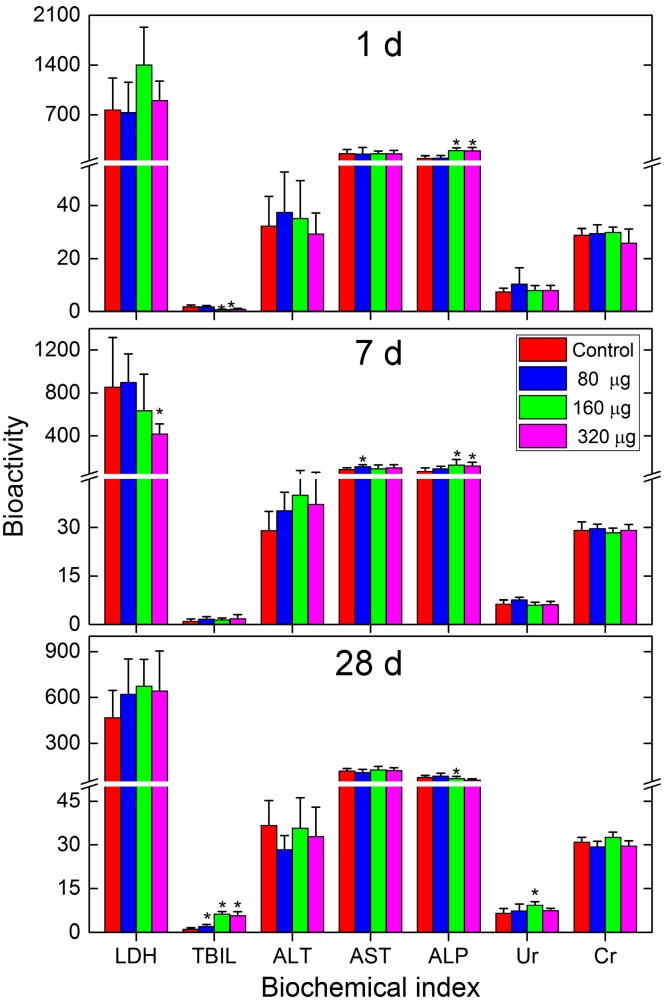
Serum biochemical parameters of the mice exposed to CNSI post intravenous exposure. Data represent means ± SD (*n* = 6). * *p* < 0.05 compared with the control group.

The serum biochemistry also confirmed the nontoxic nature of CNSI after intravenous exposure (Figure 3). Most parameters remained unchanged during the 28-day period. Total bilirubin (TBIL) showed decreases at 1 day, which recovered at 7 days and increased at 28 days. No significant increase was observed for lactate dehydrogenase (LDH) at 1 day post-exposure. A decrease of LDH level was found at 7 days for the 320 µg group. Alanine aminotransferase (ALT) and aspartate aminotransferase (AST) are very sensitive indicators for hepatic toxicity, yet only AST showed a meaningful increase at 7 days for the 80 µg group. This indicated the low toxicity of CNSI to the liver. Aspartate aminotransferase (ALP) had slight increases at 1 day and 7 days, which were eliminated at 28 days. Urea (Ur) only had an increase at 28 days for the 160 µg group. Creatinine (Cr) had no change among all groups. Again, the lack of serve changes of serum biochemistry parameters and the absence of a dose-/time-dependent effect suggested the low toxicity of CNSI after intravenous injection.

**Figure 4:**
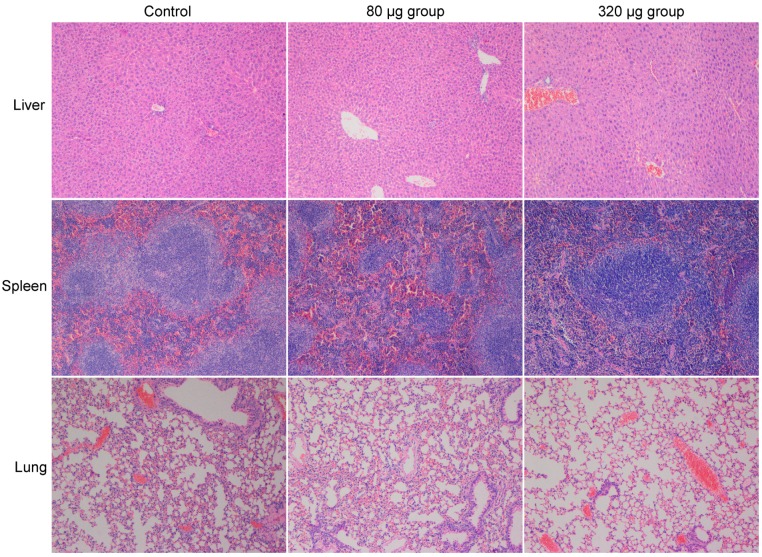
Histopathological observations of the mice exposed to CNSI at 28 days post intravenous exposure (100×).

The lack of functional changes after CNSI exposure was consistent with the histopathological observations (Figure 4), suggesting that CNSI did not induce organic damage after intravenous injection. No obvious histopathological change was observed for the liver, spleen, or lungs upon the HE staining under optical microscope at 1 day [29], 7 days [29], and 28 days. No steatosis, necrosis, or hydropic degeneration were presented in the exposed hepatic sections. Typical splenic unit and lymphocyte were presented in the spleen sections for the control group and the CNSI-exposed groups. No inflammatory cell infiltration occurred in the lung sections, which was widely observed for CNTs and graphene [21,30,31]. This was due to the high dispersibility of CNSI and small particle sizes.

**Figure 5:**
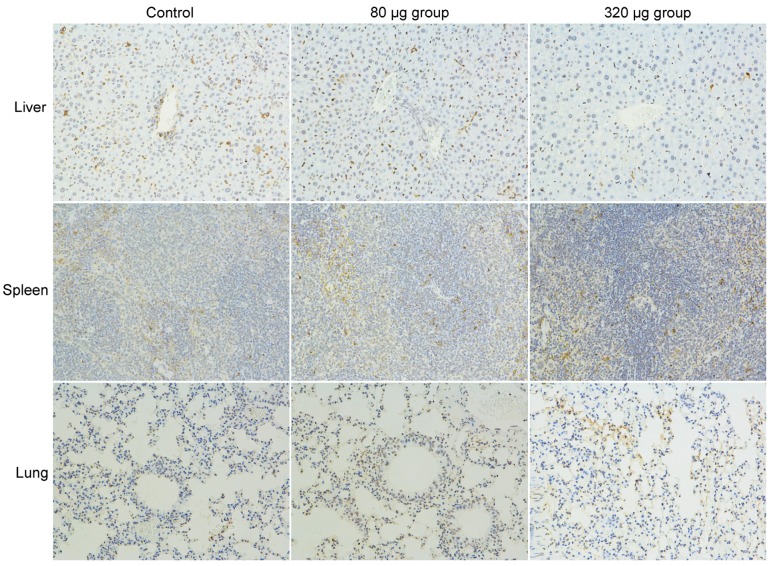
Apoptosis analyses of the mice exposed to CNSI at 28 days post intravenous exposure by the terminal deoxynucleotidyl transferase-mediated dUTP nick-end labeling (TUNEL) method (200×).

Apoptosis is a widely observed toxic symptom in the toxicity studies of carbon nanomaterials [32]. Using the TUNEL method, we checked the apoptosis levels of mice after the intravenous exposure to CNSI at 1 day [33], 7 days [33], and 28 days (Figure 5). The brown stained nuclei were counted for comparison. In the liver sections, the hepatic cells had blue cell nuclei, suggesting the absence of apoptosis. The Kupffer cells seemed to have higher apoptosis levels, but the situations were similar among the control and the CNSI-exposed groups. For the spleen, the splenic unit was stained blue, while the lymphocyte showed slight apoptosis. Again, no meaningful difference was found among the control and CNSI-exposed groups. The nuclei in the lung sections were partially stained brown, but the apoptosis levels were similar among the three groups. Overall, CNSI did not induce apoptosis in the RES organs after intravenous exposure. This was consistent with the literature results that carbon nanomaterials induced apoptosis in vitro rather than in vivo after intravenous injection [21].

**Figure 6:**
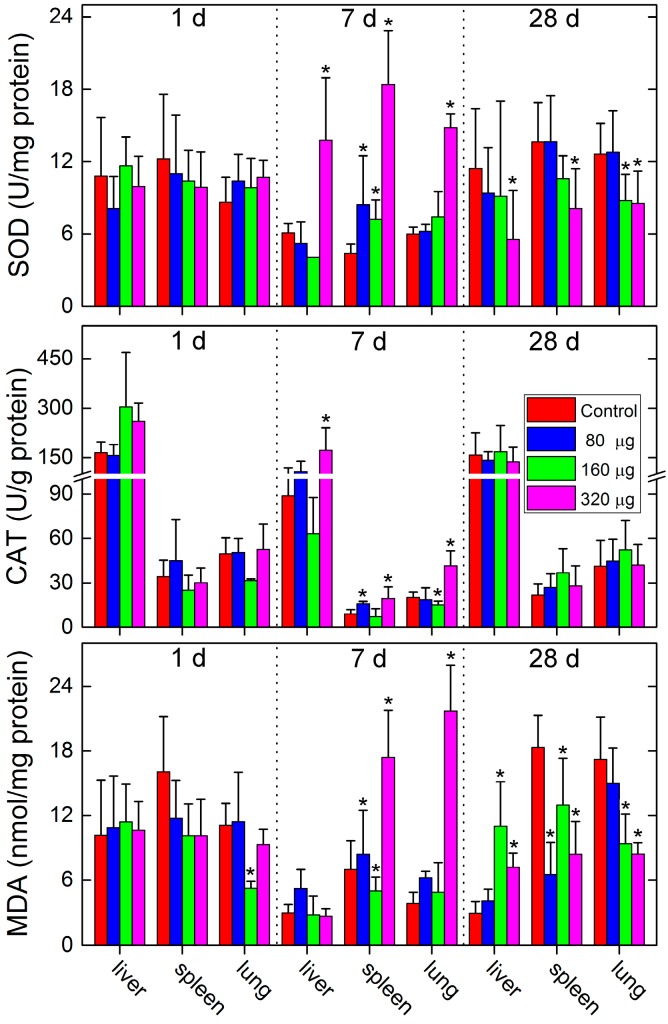
Oxidative stress levels of the mice exposed to CNSI post intravenous exposure. Data represent means ± SD (*n* = 5). * *p* < 0.05 compared with the control group.

Oxidative stress is usually regarded as the toxicological mechanism of carbon nanomaterials [34]. Oxidative stress is more sensitive than other toxicological indicators to reflect the potential hazards of carbon nanomaterials. Here, we measured the superoxide dismutase (SOD), catalase (CAT), and malondialdehyde (MDA) levels to investigate the potential oxidative stress caused by CNSI in mice (Figure 6). At 1 day post-exposure, apart from the fact that the MDA decreased in the lung of the 160 µg group, no change was observed, suggesting the absence of oxidative stress in the short term. However, oxidative stress occurred at 7 days post-exposure. The liver had higher SOD and CAT levels at 7 days in the 320 µg group, but the MDA levels remained unchanged. The spleen and lungs had increased levels of all three indicators, suggesting more oxidative stress in the spleen and lungs than in liver. The oxidative stress seemed to be alleviated at 28 days, where the levels of indicators were lower than those of the control group in the spleen and lungs. Only liver samples showed higher MDA levels in the 160 µg group and the 320 µg group. In a word, these results suggested that CNSI could incite oxidative stress in RES organs at 7 days, but the oxidative stress was alleviated at 28 days. The oxidative stress of CNSI was similar to that of other carbon nanomaterials. For example, CNTs induced oxidative stress in the liver and lung after intravenous injection [21]. Other carbon nanomaterials, such as GO and graphene quantum dots, did not induce oxidative stress after intravenous exposure [23,35]. Together with our results, the available data indicated that the oxidative stress depended on the properties of carbon nanomaterials and its rules of regulation require further investigations.

## 3. Materials and Methods 

### 3.1. Materials

Commercial CNSI (50 mg/mL) was provided by Chongqing Lummy Pharmaceutical Co., Ltd, Chongqing, China. All kits for serum biochemistry were obtained from MSKBio Science and Technology Co., Wuhan, China. All reagents for the TUNEL method were purchased from Beijing Dingguo Changsheng Biotechnology Co., Beijing, China. All kits for oxidative stress assays were obtained from the Nanjing Jiancheng Bioengineering Institute, Nanjing, China. Other chemicals were of analytical grade and used without purification.

### 3.2. Characterization of CNSI

CNSI was carefully characterized by TEM (Autoflex, Bruker, Bonn, Germany), DLS (Zetasizer Nano ZS90, Malvern Instruments, Malvern, UK), and Raman spectroscopy (Renishaw inVia plus, Renishaw, Wotton-under-Edge, UK) before use. Carbon nanoparticles without adding suspending reagents were analyzed by XPS (Axis Ultra, Kratos, Manchester, UK) and IR (Tensor27, Bruker, Germany). The C1s spectrum was analyzed by CasaXPS software (version 2.3.15, Casa Software Ltd., Knutsford, UK) following the automatic fitting protocol.

### 3.3. Animal Exposure

The animal experiments were checked and approved by the Animal Center of Southwest Minzu University. The experiments were performed strictly in accordance with the Animal Care and Use Program Guidelines of Sichuan Province, China. Institute of Cancer Research (ICR) mice (25 g) were purchased from Dashuo Experimental Animal Co., Chengdu, China, and raised in plastic cages (six mice/cage) on a 12-h light/dark cycle with ad libitum access to food and water. After the acclimation, the mice were randomly divided into groups of six mice for each CNSI exposure.

CNSI and saline were filtered with 0.22-μm filters for sterilization before use. Mice injected intravenously with 0.2 mL of saline solution in one injection were taken as the control group. The human dosage (0.71 mg/kg body weight) was multiplied with a factor for mice (9.1), resulting in the middle dosage of 6.5 mg/kg body weight for mice. Mice injected with 80 µg carbon nanoparticles per mouse (3.2 mg/kg body weight) in one injection were set as the 80 µg group. Mice injected with 160 µg carbon nanoparticles per mouse (6.5 mg/kg body weight) in one injection were set as the 160 µg group. Mice injected with 320 µg carbon nanoparticles per mouse (13 mg/kg body weight) in one injection were set as the 320 µg group. For CNSI injection, the CNSI solution was diluted with saline to ensure the injection volume of 0.2 mL. After the injection, the behaviors of mice were recorded daily and the bodyweights were measured with the interval of 3 days. Before the sacrifice at 1, 7, and 28 days, the mice were fasted for 12 h. After collecting blood, the mice were sacrificed by cervical dislocation.

### 3.4. Biodistribution of CNSI

First, we checked the histopathological samples after HE staining under an optical microscope. The heart, liver, spleen, lungs, kidneys and axillary lymph nodes of the 320 µg group at 1 day were fixed by 4% paraformaldehyde solution. The fixed samples were embedded in paraffin, thin-sectioned, and mounted on glass microscope slides using the standard histopathological techniques. The mounted sections were stained with HE for optical microscopy. Black or brown spots were carefully checked and recorded.

For Raman analyses, the tissue samples were grounded with deionized water (0.1 g tissue in 0.1 mL water) with a homogenizer. The homogenate samples were placed on glass slides and directly analyzed by the Raman spectrometer. The parameters of Renishaw micro-Raman spectroscopy system were set as: laser excitation wavelength 785 nm, 50 mW power, 50 × objective, laser spot size 50 × 2 μm^2^, 1 s collection time, data accumulation of 100 times. The baseline and smoothing were automatically performed with the software.

### 3.5. Toxicity Evaluations

For hematological analysis, the blood samples were combined with 0.1 mL 15 g/L ethylenediaminetetraacetic acid dipotassium salt (EDTA-K_2_) for anticoagulation immediately after blood collection. The hematological measurements were performed on an automatic hematology analyzer (BC-5800, Mindray Co., Shenzhen, China) following the standard protocols. For serum biochemistry analysis, the blood samples were kept at room temperature for 1 h and then centrifuged at 3338 g for 10 min to collect the supernatant. The biochemical assays were performed on a clinical automatic chemistry analyzer (Chemray360, Rayto Co., Shenzhen China) following the standard protocols. 

For histopathological observations, the samples of control and CNSI-exposed groups were fixed, sectioned, and stained by HE as described above. The samples were checked under an optical microscope. For apoptosis evaluations, the TUNEL method was adopted. The slides were prepared strictly following the manufacturer’s instructions and observed under a light microscope. The detailed protocol is presented at the official website of the manufacturer (online resource) [36].

For oxidative stress assays, each sample was minced and homogenized in 4 °C saline three times (10 s/time, intermittent for 30 s) to yield 10% (*w*/*v*) homogenate. The homogenates were centrifuged at 2225× *g* for 10 min to obtain the supernatants. Protein concentrations in the supernatants were determined according to the method of Bradford, using bovine serum albumin as the standard. The SOD, CAT, and MDA levels were analyzed following the manufacturer’s instructions using an UV-vis spectrophotometer (UV-1800, Mapada, Shanghai, China). The detailed protocols can be found at the official website of the manufacturer (online resource) [37].

### 3.6. Statistical Analysis

All data were expressed as the means of six individual samples with standard deviation (means ± SD). Significance was calculated by using the Student’s *t*-test method, where *p* < 0.05 was taken as statistically significant.

## 4. Conclusions

In summary, the accumulation and toxicity of CNSI were preliminarily evaluated after intravenous injection in mice, where CNSI was trapped in RES organs and no apparent toxicity was observed. CNSI accumulated majorly in the liver and spleen after intravenous injection, while only very small amounts were detected in the lungs. The mice behaved normally and their body weight increases were not disturbed upon exposure to CNSI. The nearly unchanged hematological and serum biochemical parameters indicated the low toxicity of CNSI in vivo, which was further confirmed by the histopathological observations and apoptosis analyses. The only hazard of CNSI was the induction of oxidative stress in mice. Overall, the low toxicity of CNSI after intravenous exposure ensures safe theranostics applications in the future and also confirms the biosafety of CNSI that entered the blood circulation during the intratumoral injection. It is hoped that our results will benefit the ongoing exploration of the clinical applications and biosafety evaluations of carbon-based nanomaterials.

## Figures and Tables

**Table 1:** Bodyweight increases of the mice exposed to CNSI post intravenous exposure. Data represent means ± SD (*n* = 6).

	Control (g)	80 µg Group (g)	160 µg Group (g)	320 µg Group (g)
Day 1	26.5 ± 1.3	25.5 ± 0.7	26.5 ± 1.5	26.3 ± 1.3
Day 7	28.7 ± 2.5	28.5 ± 1.9	29.3 ± 2.8	28.3 ± 2.4
Day 28	32.5 ± 3.1	33.4 ± 3.7	34.2 ± 4.9	33.5 ± 3.4

**Table 2:** Hematological parameters of the mice exposed to CNSI post intravenous exposure. Data represent means ± SD (*n* = 6).

	1 d				7 d				28 d			
	Control	80 μg	160 μg	320 μg	Control	80 μg	160 μg	320 μg	Control	80 μg	160 μg	320 μg
PLT (10^9^/L)	474 ± 63	486 ± 81	478 ± 126	459 ± 98	422 ± 116	578 ± 135	457 ± 138	421 ± 69	444 ± 108	332 ± 113	377 ± 63	382 ± 52
MCHC (g/L)	353 ± 52	321 ± 23	364 ± 38	339 ± 33	360 ± 50	326 ± 24	316 ± 30	313 ± 3 *	297 ± 15	304 ± 33	313 ± 22	305 ± 23
HB (g/L)	145 ± 7	155 ± 6 *	150 ± 7	156 ± 9 *	153 ± 7	154 ± 6	152 ± 4	149 ± 12	148 ± 17	142 ± 37	149 ± 4	152 ± 6
MCV (fL)	49 ± 5	56 ± 3 *	49 ± 5	52 ± 5	49 ± 6	52 ± 4	56 ± 4	57 ± 1 *	56 ± 5	58 ± 8	53 ± 5	56 ± 6
WBC (10^9^/L)	4.4 ± 1.9	5.0 ± 0.8	6.3 ± 2.5	4.5 ± 1.0	8.0 ± 3.0	5.4 ± 3.4	5.1 ± 1.9	5.0 ± 2	7.6 ± 2.8	6.1 ± 2.7	6.0 ± 2	6.9 ± 2.7
RBC (10^12^/L)	8.5 ± 0.6	8.6 ± 0.4	8.5 ± 0.4	9.0 ± 0.2	8.8 ± 0.6	9.1 ± 0.3	8.7 ± 0.3	8.4 ± 0.7	9.0 ± 0.9	8.2 ± 1.9	9.0 ± 0.4	8.9 ± 0.6
MCH (pg)	17.2 ± 0.9	18.0 ± 0.7	17.7 ± 0.3	16.9 ± 0.6	17.4 ± 0.6	17.0 ± 0.6	17.5 ± 0.4	17.8 ± 0.4	16.5 ± 1.0	17.3 ± 0.7	16.6 ± 0.4	17.2 ± 1.1
RDW (%)	18.1 ± 1.4	17.1 ± 1.8	17.9 ± 2.4	18.4 ± 2.2	17.5 ± 1.4	19.5 ± 1.3	16.4 ± 1.1	15.7 ± 1.6	16.6 ± 2.9	14.0 ± 1.2	16.0 ± 1.8	15.3 ± 1.6
MPV (fL)	7.7 ± 0.3	7.8 ± 0.3	8.0 ± 0.5	7.5 ± 0.2	7.6 ± 0.4	7.3 ± 0.2	7.4 ± 0.3	7.4 ± 0.2	7.4 ± 0.1	7.7 ± 0.4	7.8 ± 0.4 *	7.6 ± 0.3
PDW (%)	9.1 ± 0.6	9.0 ± 0.5	9.5 ± 0.8	8.7 ± 0.5	8.8 ± 0.8	8.3 ± 0.3	8.4 ± 0.5	8.4 ± 0.5	8.3 ± 0.2	8.8 ± 0.6	9.0 ± 0.8	8.7 ± 0.7

* *p* < 0.05 compared with control group. PLT, platelet; MCHC, mean corpuscular hemoglobin concentration; HB, hemoglobin; MCV, mean corpuscular volume; WBC, white blood cell count; RBC, red blood cell count; MCH, mean corpuscular hemoglobin; RDW, red cell distribution width; MPV, mean platelet volume; PDW, platelet distribution width.

## Abbreviations

CNSI	Carbon nanoparticles suspension injection
RES	Reticuloendothelial system
CNTs	Carbon nanotubes
TDLN	Tumor drainage lymph node
GO	Graphene oxide
TUNEL	Terminal deoxynucleotidyl transferase-mediated dUTP nick-end labeling
PVP	Polyvinyl pyrrolidone
TEM	Transmission electron microscopy
DLS	Dynamic light scattering
XPS	X-ray photoelectron spectroscopy
FWHM	Full width at half maximum
IR	Infrared spectroscopy
HE	Hematoxylin-eosin
HB	Hemoglobin
MCV	Mean corpuscular volume
MCHC	Mean corpuscular hemoglobin
MPV	Mean platelet volume
PLT	Platelet
WBC	White blood cell count
RBC	Red blood cell count
MCH	Mean corpuscular hemoglobin
RDW	Red cell distribution width
PDW	Platelet distribution width
TBIL	Total bilirubin
LDH	Lactate dehydrogenase
ALT	Alanine aminotransferase
AST	Aspartate aminotransferase
ALP	Alkaline phosphatase
Ur	Urea
Cr	Creatinine
SOD	Superoxide dismutase
CAT	Catalase
MDA	Malondialdehyde
ICR	Institute of Cancer Research
